# Acute Phase Peyronie’s Disease: Where Do We Stand?

**DOI:** 10.7759/cureus.67054

**Published:** 2024-08-17

**Authors:** Konstantinos Douroumis, Konstantinos Kotrotsios, Panagiotis Katsikatsos, Napoleon Moulavasilis, Evangelos Fragkiadis, Dionysios Mitropoulos, Ioannis Adamakis

**Affiliations:** 1 Department of Urology, National and Kapodistrian University of Athens, Athens, GRC

**Keywords:** male sexual health, andrology, penile curvature, acute phase, peyronie disease

## Abstract

Peyronie’s disease (PD) is a common benign condition characterized by superficial fibrosis and scar formation at the tunica albuginea of the penis, eventually leading to penile curvature. It is believed that penile micro-traumas during intercourse and subsequent activation of inflammatory processes constitute the pathogenetic basis of the disease. Routinely, PD is divided into acute and chronic phases, with pain during erection or flaccid state being the hallmark of the former. Surgical intervention should be avoided during the acute phase, as the risk of recurrence or progression of penile deformity during this stage might affect the optimal outcomes of the procedure. During this stage, many conservative treatment options have been suggested, including oral, topical, and intralesional therapies, extracorporeal shock wave therapy (ESWT), and penile traction therapy (PTT). Currently, the optimal treatment consists of a combined treatment strategy with phosphodiesterase type 5 inhibitors (PDE5Is), ESWT for pain management, PTT, and intralesional therapies. Large, well-designed randomized controlled trials (RCTs) are necessary to further elucidate the most efficient treatment option for acute phase PD.

## Introduction and background

Peyronie’s disease (PD) is a benign condition that was first described by Francois Gigot de la Peyronie more than 250 years ago [1]. Despite being known for over two centuries, PD still besets a great proportion of the male population, as manifested in a large survey of 8,000 men, where the prevalence of PD was calculated as high as 389:100,000 [2]. More recent data estimate that the condition is present in around 0.3% to 13.1% of men globally, with the prevalence increasing in specific sub-populations, such as in men operated on for radical prostatectomy. [3] Additionally, another recent survey demonstrated that the prevalence of definitive and probable cases of Peyronie’s disease in the USA is 0.7% and 11%, respectively, indicating that it is an underdiagnosed condition [4].

Peyronie’s disease is a wound-healing disorder characterized by superficial fibrosis of the penis, appearing as an inelastic scar at tunica albuginea (TA), palpable during the flaccid state [5]. The most common areas of the penis, where the plaque develops, are, in descending order, the base, the mid-shaft area, and the distal penis [6]. The main symptom of PD is penile deformity comprised of curvature during erection, accompanied by loss of flaccid penile length, tunical indentations, or hourglass deformity with erection, and buckling or penile instability on minimal axial loading, despite maximum erection [7, 8]. Except for physical symptoms, PD, which is also associated with erectile dysfunction, poses a major psychological burden on both the patients and their partners [9, 10]. The aim of this review is to investigate the latest updates on the pathophysiology of Peyronie’s disease, thoroughly examine traditional and promising treatments of the acute phase of the disease, and present the limitations and future perspectives of current research in the field.

## Review

Search strategy

A literature search was performed (December 8, 2023) using Medical Literature Analysis and Retrieval System Online (MEDLINE), PubMed, Cochrane Controlled Register of Trials (CENTRAL), Scopus, and Excerpta Medica database (EMBASE) databases. The following terms were used in the search text fields: Peyronie's disease AND (acute phase OR initial phase) AND (management OR oral therapy OR topical therapy OR surgery OR shock wave therapy OR traction therapy OR collagenase *Clostridium histolyticum* OR intralesional therapy). The search algorithm was adjusted for each database while maintaining a common overall architecture.

Published observational and interventional studies, clinical trial registries, and published conference abstracts in major urological or sexual medicine journals assessing the effect of any kind of treatment in patients with acute phase PD were included. Reviews, letters, and commentaries were excluded. In addition, we applied forward and backward citation searching by examining the reference list of all included studies and relevant reviews.

The abstracts of all articles were screened, and the full texts of all the relevant articles were examined for possible inclusion. A summary of the existing therapies for acute phase PD accompanied with their mechanism of action and probable adverse effects is provided in Table 1.

**Table 1:** Treatments for active phase Peyronie’s disease COX: cyclooxygenase; TGF: transforming growth factor; CCh: collagenase *Clostridium histolyticum*; ESWT: extracorporeal shock wave therapy; PTT: penile traction therapy; PRP: platelet-rich plasma

Treatment	Route of administration	Mechanism of action	Side effects
Non-steroidal anti-inflammatory drugs [11]	Oral/topical	Inhibit COX-1 and COX-2, inhibit fibroblast proliferation	Stomach pain, ulcers, gastrorrhagia
Phosphodiesterase-5 inhibitors [12]	Oral	Inhibit tissue remodeling by decreasing oxidative stress	Headaches, dizziness
Vitamin E [13]	Oral	Free radical scavenger	Nausea, diarrhea, fatigue
Colchicine [14]	Oral	Induces collagenase activity, inhibits tubulin in leukocytes	Nausea, diarrhea, bone marrow suppression
Pentoxifylline [15]	Oral/ perilesional	Reduces TGF-β induced collagen production	Tachycardia, rash, low blood pressure, dizziness
Coenzyme Q [16]	Oral	Suppresses TGF-β1 expression	Upper abdominal pain, nausea vomiting
Carnitine [17]	Oral	Reduces intracellular calcium concentration	Nausea, abdominal pain, mild euphoria, seizures
Omega 3 [18]	Oral	Anti-inflammatory agent	Gastrointestinal distress, fishy odor
Potaba [19]	Oral	Increase oxygen uptake, decrease serotonin concentration	Gastrointestinal distress, hypoglycemia, blue-colored skin
Tamoxifen [20]	Oral	TGF release from fibroblasts, blockade of TGF-β receptors	Hives, difficulty breathing, swelling of the face
Silymarin [21]	Oral	Anti-oxidant agent	Gastrointestinal distress
Bilberry [22]	Oral	Anti-oxidant agent	Possible allergic reactions
Propolis [22]	Oral	Anti-oxidant agent	Possible allergic reactions
Ginkgo Biloba [21]	Oral	Anti-oxidant agent	Arrhythmias, stroke, tinnitus, seizures
Superoxide dismutase [23]	Topical	Free radical scavenger	Pain at the application site
CCh [24]	Intralesional	Halts extracellular remodeling	Damage or hematoma at the injection site, fever, chills
Verapamil [25]	Intralesional	Stimulates degradation of extracellular matrix	Nausea, constipation, flu syndrome, hematoma at injection site
Hyaluronic Acid [26]	Intralesional	Restores extracellular matrix homeostasis	Itching, numbness, headache, hematoma at the injection site
Corticosteroids [27]	Intralesional	Inhibit the inflammation and the activity of phospholipase A2	Tissue atrophy, thinning of the skin
Nicardipine [28]	Intralesional	Reduction of glycosaminoglycan synthesis and blockade of collagen production	Rash, ecchymosis, pain hematoma at the injection site
Interferon-alpha 2b [29]	Intralesional	Decrease in fibroblast and collagen proliferation	Flu-like symptoms, hematoma in injection site
ESWT [30]	Extra-corporeal	Mechanical stimulus promoting biological healing	Pain, red skin, numbness, hyperesthesia
PTT [31]	NA	Tissue remodeling through mechanotransduction	Discomfort, pain, swelling, numbness
PRP [32]	Intralesional	Extracellular matrix remodeling	Bleeding, tissue damage, local Infection, nerve injuries

Pathophysiology and natural history of the disease

The pathogenesis of the disease is not completely elucidated, but the most widely acceptable theory accuses micro-trauma and its subsequent inflammatory response of leading to fibrosis with reduction of elasticity and aberrant collagen deposition [33, 34]. Histologically, PD is characterized by immoderate production and accumulation of collagen and other components of the extracellular matrix (ECM), eventually leading to the formation of inelastic penile plaques [35]. It has already been well established that transforming growth factor (TGF-β1) plays a crucial role in the pathophysiology of the disease. More specifically, the overexpression of this cytokine promotes the differentiation of fibroblasts into myofibroblasts, which can evade apoptosis, leading to the accumulation of excessive ECM components, especially collagen types I, III, and IV [36].

Given that the ECM serves not only as a supporting structure for the cells but also as a coordinator for multiple signaling pathways by binding transmembrane receptors and soluble ligands, its modifications during repeated micro-traumas and consequent inflammation may play a role in PD development. This ECM reorganization induced by topical hypoxia, low pH, accumulation of reactive oxygen species, and production of multiple cytokines and growth factors leads to the release of latent TGF-β1 [37, 38]. At high levels of TGF-βa, redox imbalance occurs due to inhibition of antioxidant enzymes including glutathione peroxidase, glutathione synthetase, and catalase [39]. As a consequence, plasminogen activator inhibitor (PAI-1) expression is enhanced, and fibrinolysis is undermined [40]. Simultaneously high concentrations of TGF-β1 contribute to an imbalance between matrix metalloproteinases (MMP) and tissue inhibitors of matrix metalloproteinases (TIMP), reinforcing the fibrosis procedure [41]. Furthermore, other major activators of the TGF-β cascade are connective tissue growth factor and angiotensin II, through suppression of Smad7 and increase of the expression of thrombospondin-1, respectively [42, 43].

Besides TGF-β signaling, other cellular transduction pathways seem to be associated with excess myofibroblast proliferation, chronic inflammation, and fibrosis present in PD. These include Wnt/b-catenin, Notch, hedgehog, hippo, Rho/ROCK, PI3K/Akt/mTOR, p38 MAPK/ERK, JAK-STAT, and NF-kB signal pathways [35]. Additionally to all the interacting molecular pathways, specific morphological differences in TA, and especially a single-layered TA, can be more prone to disturbance due to mechanical forces applied to the penis during sexual intercourse [44].

Now, regarding the genetic background of the disease, the disrupted expression of TGF-β1 has been associated in a study with a point missense mutation in the TGF-β1 gene due to a specific single nucleotide polymorphism (rs1800471; G915C) [45]. Moreover, karyotyping and analysis of fibroblasts’ genetic samples derived from PD revealed both chromosomal and gene abnormalities related to the disease in loci responsible for ECM reorganization and cytokine production [46]. Characteristic examples include alterations in Wnt, Fas, and Bcl-2 expression. These gene modifications can maintain the fibrotic procedure by giving the myofibroblasts the ability to evade apoptosis [47-49]. Also, epigenetic mechanisms, with the main representative being histone deacetylase (HDAC) modification, seem to play a significant role in disease pathophysiology, making it a potential target for future therapies [50].

The natural history of PD is routinely divided into active and stable phases. The hallmarks of the active or acute phase are penile deformity, which might be combined with a soft plaque and pain in an erect and/or flaccid state. The severity of the penile curvature might increase (21%-48%), not change (36%-67%), or decrease (3%-13%). The acute stage of the disease commonly lasts for six to 18 months, with the pain improving or even alleviated in the majority of patients within 12 months of PD presentation. On the other hand, the stable phase is characterized by stability in penile curvature for at least three to six months and improvement or resolution of pain. During the chronic phase, other deformities, such as hourglass deformities and hinge effects, as well as shortening of the penis, can be present [7,51,52]. Except for the distinction of acute and stable phase using clinical criteria, a novel approach where neutrophil-to-lymphocyte (NLR) and platelet-to-lymphocyte ratio (PLR) calculation can further assist the urologist [53]. The definitive treatment of PD is the surgical intervention, which, however, should be avoided during the acute phase, as the risk of recurrence or progression of penile deformity during this stage might affect the optimal outcomes of the procedure [54,55]. Thus, taking into account the psychological distress of the patients, as well as the pain characterizing the acute phase of the disease, these patients are in urgent need of treatment modalities that are equally effective and safe as the surgical procedure [56].

Treatment strategies

**Figure 1:**
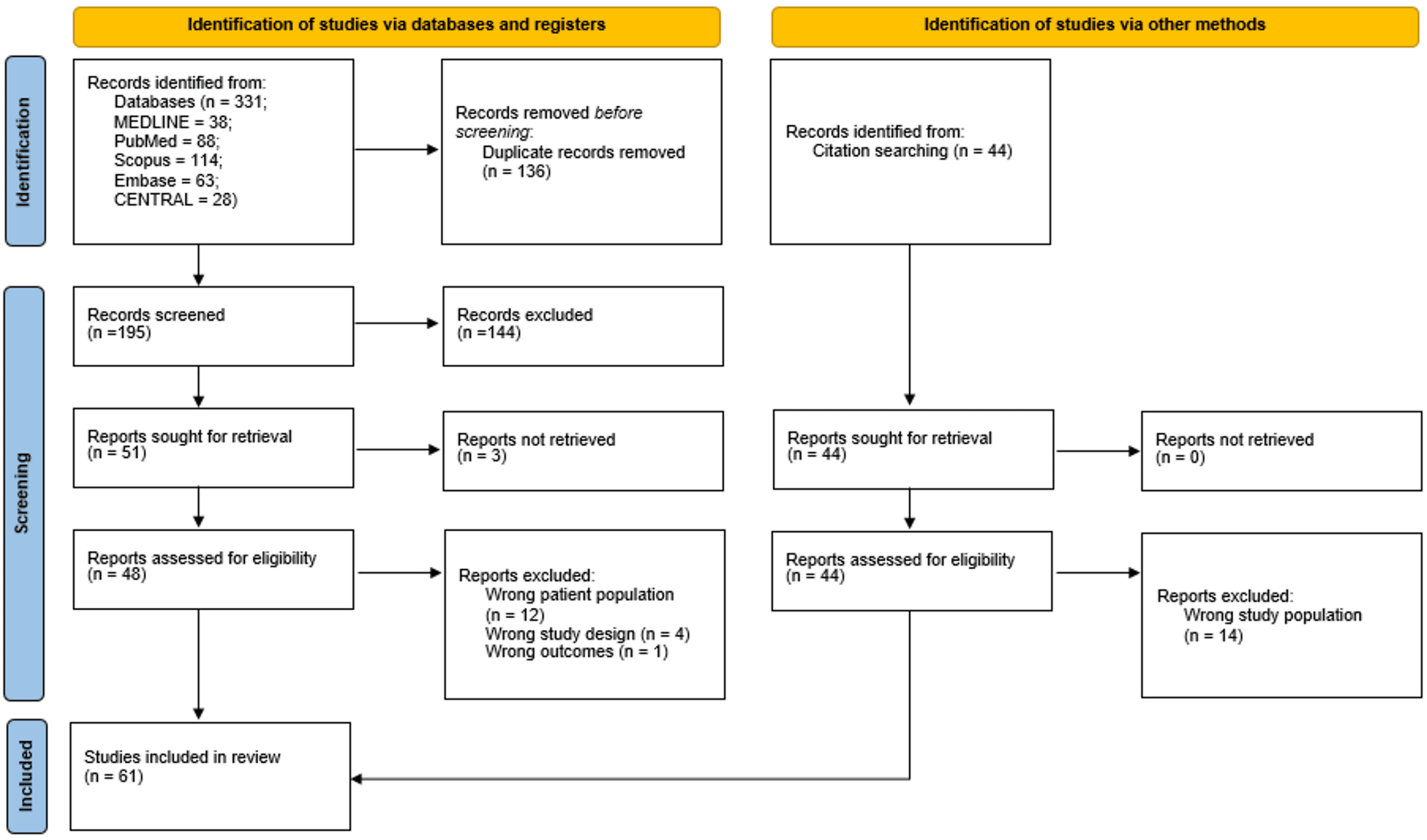
A PRISMA flow diagram summarizing the selection process [57]. MEDLINE: Medical Literature Analysis and Retrieval System Online; Embase: Excerpta Medica database; CENTRAL: Cochrane Controlled Register of Trials; PRISMA: Preferred Reporting Items for Systematic Reviews and Meta-Analyses

A flow diagram of the selection process is presented in Figure 1 [57]. We initially identified 331 papers, and after deduplication, 135 articles were considered eligible for title-abstract screening. Subsequently, 51 publications were selected for full-text screening, and, finally, 31 of them met the inclusion criteria and were included. Furthermore, the references of the included studies and references from other relevant studies from high-impact journals were hand-searched, and 30 papers that were lost from the initial literature search were included too.

Oral Therapies

Systemic administration of therapeutic regimens by the oral route was among the first non-surgical approaches to PD management described in the literature. For instance, almost five decades have passed since the publication of the earliest data with respect to the effect of procarbazine on PD progression [58, 59]. Since then, many oral agents have been proposed as hypothetically efficient treatment options.

Colchicine

The first use of colchicine in the treatment of PD was published in 1994. The hypothesis was that colchicine was already known to induce collagenase activity and decrease collagen synthesis, so it could be used in the treatment of PD. The results of this study were promising [14]. An early study showed that colchicine could be effective in the early stage of PD, especially in pain resolution [60]. Similar results were observed in a cohort of patients under 40 years of age [61]. A subsequent randomized double-blind study showed colchicine is no better than placebo [62]. Also, a study examining the alteration of penile curvature under colchicine showed conflicting results [63].

Potassium para-aminobenzoate (potaba)

Potassium para-aminobenzoate (Glenwood, LLC, Englewood, NJ) was first used in the treatment of PD in 1959 [19]. Promising results, concerning penile pain, plaque size, and curvature, in multiple studies before 2000 established the use of potaba in the treatment of acute phase PD [64-66]. A prospective randomized placebo-controlled study showed that potaba had a significant plaque-related effect. Specifically, mean plaque size decreased from 259 mm2 to 142 mm2 in the Potaba arm. In the placebo arm, the plaque size increased from 259 mm^2^ to 303 mm^2^ after six months, but decreased to 233 mm^2^ after 12 months, with the difference between the groups being significant (p = 0.042). On the contrary, potaba showed no effect in improving pre-existing curvature or decreasing penile pain [67]. In a retrospective study comparing Potaba as monotherapy with a combination therapy of tamoxifen, L-carnitine, and phosphodiesterase type 5 inhibitors (PDE5Is), potaba monotherapy improved plaque size and pain without statistical differences with the combination therapy, but had high drop-out rates, with 68.2% of patients discontinuing treatment within 12 weeks. Also, combination therapy showed better results in successful sexual intercourse and in the avoidance of the need for surgery [68].

Vitamin E

Scardino and Scott (1949) were the first to report the use of vitamin E (tocopherol) in the treatment of PD, making it the oldest oral therapy in the management of the disease [13]. It has antioxidant, anti-inflammatory, and anti-fibrotic properties, and it is believed to reduce the collagen deposits within the tunica albuginea. The first double-blinded, placebo-controlled study, published in 1983, showed no significant differences regarding improvement in plaque size or curvature [69]. These results were confirmed by later studies, analyzing vitamin E alone as well as in comparison with propionyl-L-carnitine [70, 71]. On the other hand, interesting results were extracted by studies examining the efficacy of vitamin E in combination with other non-surgical treatments, indicating that vitamin E might be used as part of a multimodal therapy [72, 22].

Tamoxifen

Treatment with tamoxifen showed potential based on an earlier uncontrolled trial, with improvements in penile pain and plaque shrinkage [20]. These results were questioned in a well-controlled study with patients only in the acute phase of the disease that demonstrated that treatment with tamoxifen did not improve pain, curvature, or plaque size [73]. However, the combination of tamoxifen with a PDE5I seemed to halt the progression of patients with acute PD compared to the control group receiving either vitamin E or no drug. More specifically, in the investigation group, more patients experienced an improvement in penile curvature (26.5% vs. 7.3%), and fewer patients displayed worsened curvature (12.7% vs. 26.8%) [74].

Phosphodiesterase type 5 inhibitors

Phosphodiesterase type 5 inhibitors were first used in the treatment for PD in 2003, as it was hypothesized that through the inhibition of TGF-β1, they promote the reduction of collagen deposition [12]. A retrospective clinical trial was conducted in 2011, with the aim of examining if the administration of PDE5is can promote the resolution of isolation septal scars without evidence of penile deformity. The results were encouraging, as the tadalafil group reported a higher International Index of Erectile Function (IIEF-5) score, and resolution of the septal scar was observed in 24 patients (69%) compared to three patients (10%) in the control group [75]. Also, in a head-to-head comparison with Vitamin E, 12 weeks of sildenafil administration showed significantly better outcomes in the IIEF-5 scores and pain reduction compared to the vitamin E group [76]. A recently published retrospective study examined the effect of daily tadalafil 5 mg administration in patients with acute phase PD and erectile dysfunction. This study showed that tadalafil administration in the acute phase reduces the curvature progression and improves erectile function and PD-related symptoms [77]. Finally, another recently published study showed that the addition of 50 mg daily sildenafil to pentoxifylline-colchicine combination therapy has significantly better results in the degree of curvature, pain, and erectile function compared to pentoxifylline-colchicine combination therapy alone [78]. These results, along with the absence of significant side effects of PDE5Is, have led to the widespread usage of PDE5Is in the management of the acute phase of PD.

Pentoxifylline

Pentoxifylline has been used in the treatment of acute phase PD in combination treatment with other antioxidants and topical diclofenac. Multimodal treatment with pentoxifylline was efficacious in the treatment of acute phase PD, as decreases in plaque volume, penile curvature, and pain were observed, along with recovery of penile rigidity, in contrast to the control group. An interesting result was that the treatment appeared to be more effective when pentoxifylline was administered both orally and by penile injection [15].

Coenzyme Q (CoQ)

Coenzyme Q exerts strong suppression on oxidative stress and has been recommended as a therapeutic option in active-phase PD. Poor evidence is available to support this hypothesis, with only one randomized controlled trial (RCT) demonstrating that daily doses of CoQ for six months resulted in greater improvement in sexual function, plaque size, and penile curvature compared to the placebo control group [16].

Acetyl-L-carnitine

Acetyl-L-carnitine, which is the precursor of acetylcholine, as an oral regimen has proven useful in degenerative diseases by increasing free radical metabolism and preventing intracellular calcium overload. A small 2001 RCT comparing the efficacy of orally delivered carnitine versus tamoxifen demonstrated that the former is superior regarding pain improvement, curvature decrease, and disease progression, markedly without causing any adverse effects [17]. 

Nonsteroidal anti-inflammatory drugs

As pain is the main symptom in acute-phase PD, nonsteroidal anti-inflammatory drugs may be offered for penile pain management. Treatment efficacy should be monitored periodically with an assessment of pain levels [11].

Regional therapies

The first promising results about the efficacy of topical regimen application in acute phase PD were derived from an uncontrolled phase 2 clinical trial including 13 patients. In this study, the regional use of recombinant human (rh) Cu-Zn superoxide dismutase (SOD) was able to reduce pain in all patients (p<0.05), without causing any local or systemic side effects [23]. These results constituted the basis for a phase 3 RCT, which verified the greater effectiveness of rhSOD in reducing pain in the intervention group against the placebo group (52.6% vs. 20%, respectively, p = 0.017). However, after 12 months of follow-up, it had no significant impact on plaque size or penile curvature [79].

Another RCT examined the efficacy of a gel containing not only SOD but also nicardipine and emu oil (H-100 gel). This combination of regimens being applied for six months in 22 patients managed to significantly improve penile curvature (40.8% reduction, p = 0.0014), stretched penile length (22.6% increase, p = 0.0002), and mean pain level (85.7% reduction, p = 0.004). Regarding the safety of the regime, a self-resolving rash in two patients and a more persistent rash in one patient were observed [80].

Collagenase Clostridium histolyticum (CCh)

Collagenase *Clostridium histolyticum* is the oldest intralesional therapy and also the most popular in managing acute phase PD. In a study conducted by Cocci et al. in 2020, 74 patients were enrolled with acute phase PD and a mean curvature of 41.1o ± 12ο. All patients received a single dose of intralesional CCh. The mean follow-up was six months. There were statistically significant results concerning the Visual Analog Scale (VAS) score both at rest and during intercourse and in terms of IIEF-5 score improvement [24].

In a retrospective study with 162 patients, Nguyen et al. investigated the efficacy of CCh in the active phase. In this single-center study, 36 patients (22%) were found to be in the active phase and the remaining 126 (78%) were in stable phase. The mean number of cycles for all patients was 3.2. After the treatment, there was a statistically significant improvement in curvature from a mean of 57.7^o^ to 41.9^o^. There was no statistical difference between the two groups concerning the final change in curvature, the IIEF-5 score, or the stretched penile length. Moreover, there was no difference in treatment-related adverse events between the two groups (p = 0.356). This study indicated that CCh is efficient and safe both in the acute and stable phases of PD [81].

In a larger and multi-institutional analysis conducted by Nguyen et al., 918 patients were enrolled in the analysis, of which 134 (14.6%) were found to be in the active phase and the remaining 784 (85.4%) in the stable phase. Patients were standardized to an Investigation of Maximal Peyronie's Reduction Efficacy and Safety Studies (IMPRESS) protocol schedule of CCh injections with small variations among institutions. Penile modeling was initiated 24-72 hours after the second injection of each cycle. There was no difference in pre-treatment patient characteristics between the two groups. After treatment, there was no statistically significant difference between the acute and stable phases in the final change in curvature (13.5^o^ vs. 15.6^o^, p = 0.09). In addition, there was no difference in the frequency of treatment-related adverse events between the acute phase and the stable phase. The authors concluded that the number of cycles is the only predictive factor of improvement of curvature and that CCh therapy is efficient and safe in the acute phase of PD [82].

In the most recent study, Hu et al. retrospectively assessed 330 patients from a single institution who received intralesional CCh for acute phase PD. There were two definitions of acute phase used: (1) acute phase duration ≤6 months and (2) acute phase duration ≤12 months with penile pain. Of 330 patients, 229 qualified for analysis because they had both pre-and post-treatment erect penile goniometry. The mean follow-up time was 10.5 ± 4.2 months. Two separate analyses were conducted for definition 1 and definition 2 of the acute and chronic phases of PD. Sixty-five patients (28%) met the criteria for definition 1 of the acute phase, 37 (16%) for definition 2, and 76 (33%) for either definition. There was no difference in percent change in penile curvature between acute and chronic phases using definition 1 (p = 0.89), definition 2 (p = 0.43), or either (p = 0.96). Additionally, no statistically significant difference was found in the frequency of complications between the groups of acute and chronic phases under either definition (p>0.05). Conclusively, intralesional CCh was effective and safe in the acute phase of PD using multiple definitions [83].

Calcium Channel Antagonists

Calcium channel antagonists, and more specifically verapamil, were introduced in PD treatment in 1994 by Levine et al., who first reported that verapamil injections can soften and reduce plaques in the penis [25]. Since then only a few studies have evaluated the effectiveness of intralesional verapamil injections (IVI) in patients with active phase PD, leading to not adequately convincing results. The majority of patients treated with IVI monotherapy experienced a stabilization of the curvature but exhibited an increase in stretched penile length and an improvement in penetration ability [84,85]. However, both the small sample and the short follow-up period do not permit the extraction of reliable conclusions. In contrast with verapamil, which is a non-dihydropyridine (DHP), nicardipine is a DHP and is reportedly more efficient in glycosaminoglycan biosynthesis and ECM production in vitro [28]. In vivo, an RCT assessed the potency of nicardipine using 0.9% saline injections as a control and revealed a significant benefit of nicardipine in pain, erectile function, and plaque size, but not in penile curvature [86].

Hyaluronic Acid (HA)

Hyaluronic acid has been known to reduce scar formation and disrupt inflammatory and oxidative stress processes. In this context, its probable positive effect on the inflammatory and pre-fibrotic states of PD was investigated in a single-arm prospective study including 65 patients receiving 10 weekly injections with HA. This study demonstrated a statistically significant improvement in plaque size, curvature, erectile function, and pain and constituted the basis for further, more reliable research [26]. In fact, HA was compared with a commonly offered regimen in PD, meaning IVI. The outcomes were contradictory, with a non-randomized prospective study concluding that HA is superior to IVI as regards plaque size, curvature, pain, erectile function, and patient satisfaction, while an RCT detected no significant intergroup differences in these measures except for curvature and patient satisfaction. None of these two studies reported any injection-site ecchymosis or hematomas [87,88]. It is highly important to underline that none of the aforementioned studies followed the same injection protocol, with the duration varying from eight to 12 weeks. As soon as there was controversy about the true effect of HA in acute PD, the simultaneous administration of oral and intralesional HA was examined against intralesional HA monotherapy and proved to be more effective with respect to plaque size, penile deviation, pain, erectile function, and patients’ impression of improvement [89].

Corticosteroids

Taking into account that during the early stages of PD inflammatory processes are at the forefront, a direct intraplaque administration of a potent and easily accessible anti-inflammatory agent, such as methylprednisolone, hypothetically would be beneficial for this condition. Actually, this hypothesis was not completely verified in the setting of a prospective single-arm study where eight-weekly intralesional injections of methylprednisolone failed to accomplish a statistically significant decrease in penile curvature and IIEF-5 score, whereas managed to improve plaque size and all PD Questionnaire aspects [27].

Platelet-Rich Plasma (PRP)

Platelet-rich plasma, which is produced by a fraction of autologous blood enriched with platelets, appears to have a regulatory role in inflammation and extracellular matrix remodeling. Its efficacy on PD was investigated in a small pilot study including 17 patients with PD in the early stages who underwent a series of three injections every 15 days. The study manifested that PRP injections are both safe and efficient, leading not only to a significant penile curvature decrease but also to an improvement in physical and psychological symptoms as well as discomfort [32]. A better context for efficacy assessment was provided by a prospective study that compared PRP injections with placebo injections of 0.9% saline. It was reported that the intervention group experienced a statistically significant improvement in penile deviation, plaque size, plaque softening, pain, and IIEF-5 score. Last but not least, both studies did not observe any significant side effects [90].

Extracorporeal Shock Wave Therapy (ESWT)

Extracorporeal shock wave therapy was first applied in a population consisting of 26 patients with a PD duration of less than three months. Despite the small population, ESWT seemed to have a great analgesic effect and improve erectile function even after only one session [30]. In a more recent randomized controlled double-blind trial, where the therapeutic plan included four weekly sessions of ESWT, it was manifested that ESWT can be an efficient treatment modality in terms of erectile pain, erectile function, and overall quality of life. Moreover, even if no statistical significance was reached, ESWT achieved stability in penile curvature and plaque size in contrast with the placebo group, which experienced disease progression [91]. The results emerging from a recent large retrospective study point in the same direction, demonstrating that ESWT is significantly associated with pain alleviation and erectile function improvement. However, ESWT seemed to have no effect on the natural course of the disease with respect to penile curvature, which worsened at first and then stabilized [92]. In fact, the same efficacy profile of ESWT in active-phase PD arose from two studies that designate ESWT only as a potent analgesic modality [93, 94]. It is important to highlight that no significant or long-term adverse effects were observed except for mild local discomfort.

Electromotive Administration

It has been hypothesized that electromotive administration can provide a more efficient drug delivery to the TA, along with less discomfort due to multiple injections. This hypothesis was investigated by a randomized study with 60 patients during the acute phase of PD comparing the administration of verapamil and dexamethasone either electromotively or intralesionally without including side effects in its endpoints. This study was only able to provide indications and no reliable conclusions about the superiority of electromotive administration in improving penile curvature, plaque size, erectile function, and pain [95]. Additionally, another larger RCT set the electromotive administration of either verapamil and dexamethasone or lidocaine side by side. The lidocaine arm exhibited equal results with the intervention arm regarding pain reduction, while intergroup differences in plaque size and penile deviation were statistically significant in favor of the verapamil and dexamethasone combination. Besides a transient erythema at the sites of the electrodes, no other complications were reported [96].

Penile Traction Therapy (PTT)

The sole study investigating the effect of PTT in patients with acute phase PD was a non-randomized prospective controlled trial including 95 patients. More specifically, 55 patients who followed a therapeutic scheme, which demanded the use of a traction device at least six hours daily for six months, were compared with 41 patients who did not agree to accept any therapeutic option during the acute phase. This study manifested that PTT achieved a mean decrease of 20o (p< 0.05) from baseline curvature, an increase in stretched penile length and flaccid girth (p = 0.03), and a decrease in mean VAS pain score from 5.5 to 2.5 at six months of treatment (p<0.05). Also, PTT decreased the percentage of patients who were not able to penetrate (from 61.8% to 20% at six months, p< 0.03). On the other hand, in the no-intervention group, penile curvature, pain during erection, and the percentage of patients unable to penetrate all worsened to a statistically significant degree. Regarding the safety of PTT, no major complications were reported except for discomfort and erythema of the balanoprepucial sulcus [31].

Combination

The fact that monotherapies are not capable of improving all aspects of the PD clinicopathological spectrum has motivated many teams to seek out more efficient treatment strategies in combinations of regimens. The combination of oral agents and intralesional therapy has been well scrutinized, leading to contradicting results. Interferon-2b injections alongside vitamin E administration proved no statistically significant improvement in subjective parameters while merging verapamil injections with oral tadalafil resulted in an erectile function enhancement and plaque size shrinking despite no effect on penile curvature [29,97].

In addition, it was hypothesized that tadalafil can have synergistic effects not only with verapamil but also with ESWT. This hypothesis was tested in an RCT that performed a head-to-head comparison between tadalafil accompanied with ESWT and ESWT monotherapy. None of these two therapeutic strategies achieved a statistically significant improvement in pain and curvature degree after 24 weeks, and no intergroup superiority regarding pain and penile curvature was observed. However, it was manifested that adding tadalafil to ESWT improved in a statistically significant degree the 24-month IIEF-5 and Quality of Life (QoL) scores [98]. Similar conclusions arise from a small randomized controlled study where 20 patients were randomly assigned to either low-dose daily tadalafil for three months or to four low-energy ESWT sessions. Both of these treatment modalities improved erectile function during the acute stage of the disease, but penile curvature and plaque size were not significantly altered in both groups [99].

Given that PTT has offered some encouraging results even as monotherapy in acute PD, it was also inspected as part of a multi-treatment strategy. First of all, a large retrospective study with 170 patients revealed that intralesional verapamil injections, PTT, and antioxidants combined achieved improvements in mean penile curvature and stretched penile length but without any statistical significance, but they were adequate to significantly increase the mean IIEF-5 score. However, it is important to underline that due to a lack of motivation and skepticism about results, many patients refused to complete the injection and traction therapies [100]. On the other hand, an RCT that compared an arm receiving PTT and antioxidants with a control arm undergoing only verapamil injections concluded that combination treatment managed to significantly reduce both pain and penile curvature, in contrast with intralesional verapamil therapy, which failed to offer any benefit. Sadly, none of the two arms showed any improvement regarding plaque size, penile length, or IIEF-5 scores [101].

Current limitations and future perspectives

The differentiation between acute and stable phases of PD is based on clinical criteria. However, among the guideline panels, there is no well-accepted time point established as a cut-off for acute phase PD, while regarding the definition of stable phase PD, there is both agreement and discrepancy [102]. Moreover, the lack of consensus with respect to definitions of the disease phases is profound in PD intervention studies found in literature, where heterogeneous criteria, including total symptom duration, presence of pain, or duration of stable symptoms, are commonly utilized [103]. This comes to an agreement with our own experience during the conduct of this review, where no consistent definition was used across the studies, leading to a significant degradation of their internal validity and quality of evidence. Based on these discrepancies in definitions, additional tools might assist clinicians in distinguishing the two phases of the disease, such as NLR and PLR or ultrasound imaging [6,53].

One treatment modality that seems to have promising results is PTT, which can be effective in penile curvature, pain, and erectile function improvement both as monotherapy or combined with oral and intralesional therapies [31,100,101]. Although there is no standardized protocol for traction device usage, given that they should be used for at least three hours daily, in the particular studies the utilization patterns were adequate [104]. Moreover, traction therapy demands the tolerance of prolonged forces on the penis daily for six months in most studies. The long therapy duration has been proven to be a factor in poor compliance and can alter the subjective opinion of the patient about the therapy’s effectiveness [105]. Furthermore, it is not only the compliance rates an issue regarding reliable study design but also the short follow-up periods. For example, IIEF-5 scores cannot be calculated three months after treatment considering that IIEF-5 evaluates erectile function during the last six months. Last but not least, the prolongation of follow-up will provide precious results regarding the duration of response and long-term adverse effects.

According to European Association of Urology (EAU) and American Urological Association (AUA) guidelines, it is suggested, with a low level of evidence, that only intralesional CCh or interferon and PTT can be offered as treatment options to the patient, while oral regimens are not proposed as treatment options due to a lack of either evidence or efficacy, with the sole exception being PDE5is. In addition, ESWT can be a useful tool in order to improve penile pain [67,87,89,106]. However, due to the recent withdrawal of CCh from many countries, an increase in interest in combination therapies has emerged [107]. The lack of high-quality RCTs, where only recommended regimens are combined, stands in the way of reaching a consensus on reliable combination strategies [18]. Additionally, the absence of high-quality evidence does not only refer to combination treatments but also to oral therapies. For example, given that the only study investigating the efficacy of CoQ was published by an author with two retracted RCTs regarding other orally administered compounds, there are some concerns with respect to the integrity and reliability of the study [108,109].

The pathophysiology as well as the molecular paths being involved in the pathogenesis of the disease are not completely elucidated. This can be partially explained by the fact that many relevant translational research studies have included animal models and cultured PD plaques, which has significant limitations, and additionally by the difficulty to superimpose knowledge about other fibrotic diseases on PD due to heterogeneity of fibroblasts and ECM in different tissues [110]. In the context of broadening our knowledge about molecular cascades responsible for PD development, the yes-activated protein (YAP) was investigated. The YAP pathway is significantly activated in PD fibroblasts and can be inhibited by Verteporfin, a molecular inhibitor of the hippo-YAP pathway, which can constitute a promising future therapy [111]. Last, but not least, based upon the innovative insights about PD pathophysiology, *Scutellaria baicalensis* extract, a flavonoid that downregulates monocyte chemoattractant protein 1 (MCP-1) administered to active phase PD patients by oral route for six months, posed a significant impact in pain decrease, plaque volume reduction, and need for surgical repair of the curvature compared with the untreated control group [112].

## Conclusions

Acute phase PD is a challenging entity with respect to both diagnosis and management. The treatment strategy has shifted from traditional oral therapies to new combination modalities. The majority of these multimodal treatments include CCh, PPT, and PDE5Is. Large, well-designed RCTs are necessary to establish a standard of care in the treatment of this condition. Optimistically, as more light is shed on the pathophysiology of the disease, new agents might emerge and solve the acute phase PD treatment riddle.
